# Altered Adipose Tissue DNA Methylation Status in Metabolic Syndrome: Relationships Between Global DNA Methylation and Specific Methylation at Adipogenic, Lipid Metabolism and Inflammatory Candidate Genes and Metabolic Variables

**DOI:** 10.3390/jcm8010087

**Published:** 2019-01-13

**Authors:** Daniel Castellano-Castillo, Isabel Moreno-Indias, Lidia Sanchez-Alcoholado, Bruno Ramos-Molina, Juan Alcaide-Torres, Sonsoles Morcillo, Luis Ocaña-Wilhelmi, Francisco Tinahones, María Isabel Queipo-Ortuño, Fernando Cardona

**Affiliations:** 1 Unidad de Gestión Clínica de Endocrinología y Nutrición del Hospital Virgen de la Victoria, Instituto de Investigación Biomédica de Málaga (IBIMA), Universidad de Málaga, 29010 Málaga, Spain; danie__cc@hotmail.com (D.C.-C.); l.s.alcoholado@gmail.com (L.S.-A.); brunoramosmolina@gmail.com (B.R.-M.); juan.alcaidetorres@gmail.com (J.A.-T.); sonsoles75@gmail.com (S.M.); fjtinahones@uma.es (F.T.); fernandocardonadiaz@gmail.com (F.C.); 2 Centro de Investigación Biomédica en Red de Fisiopatología de la Obesidad y la Nutrición, CIBERobn, 28029 Madrid, Spain; 3 Unidad de Cirugía Metabólica, Hospital Clínico Virgen de la Victoria, 29010 Málaga, Spain; luisowilhelmi@hotmail.com; 4 Unidad de Gestión Clínica de Oncología Médica del Hospital Virgen de la Victoria, 29010 Málaga, Spain

**Keywords:** epigenetics, DNA methylation, adipose tissue, metabolic syndrome

## Abstract

Metabolic syndrome (MetS) has been postulated to increase the risk for type 2 diabetes, cardiovascular disease and cancer. Adipose tissue (AT) plays an important role in metabolic homeostasis, and AT dysfunction has an active role in metabolic diseases. MetS is closely related to lifestyle and environmental factors. Epigenetics has emerged as an interesting landscape to evaluate the possible interconnection between AT and metabolic disease, since it can be modulated by environmental factors and metabolic status. The aim of this study was to determine whether MetS has an impact on the global DNA methylation pattern and the DNA methylation of several genes related to adipogenesis (PPARG, PPARA), lipid metabolism (RXRA, SREBF2, SREBF1, SCD, LPL, LXRb), and inflammation (LRP1 C3, LEP and TNF) in visceral adipose tissue. LPL and TNF DNA methylation values were significantly different in the control-case comparisons, with higher and lower methylation respectively in the MetS group. Negative correlations were found between global DNA methylation (measured by LINE-1 methylation levels) and the metabolic deterioration and glucose levels. There were associations among variables of MetS, BMI, and HOMA-IR with DNA methylation at several CpG positions for the studied genes. In particular, there was a strong positive association between serum triglyceride levels (TG) with PPARA and LPL methylation levels. TNF methylation was negatively associated with the metabolic worsening and could be an important factor in preventing MetS occurrence according to logistic regression analysis. Therefore, global DNA methylation and methylation at specific genes related to adipogenesis, lipid metabolism and inflammation are related to the etiology of MetS and might explain in part some of the features associated to metabolic disorders.

## 1. Introduction

Metabolic syndrome (MetS) is a cluster of metabolic alterations which altogether increase the risk of suffering diabetes, cardiovascular disease and cancer [1,2]. These metabolic risk factors include high glucose, TG, and blood pressure, low HDL cholesterol (HDL-c) and greater waist circumference [1]. Genetic and lifestyle factors have been shown to be important for the development and etiology of MetS [3]. 

DNA methylation is one of the epigenetic mechanisms present in the cell. It consists of the addition of a methyl group to the carbon 5 of a cytosine pyrimidine ring next to a guanidine nucleotide, which is commonly called a CpG residue. CpG islands (CpGi) are regions composed of a high incidence of repetitions in CpG di-nucleotides inside the genome. These CpGi are usually located in or near gene promoters and are usually hypo-methylated, while gene bodies and other intergenic regions are usually hypermethylated [4]. This hypomethylation at CpG promoter regions assures that the transcription starts at the beginning of the gene, avoiding truncated transcripts [5,6]. 

On the other hand, environmental and lifestyle factors are related to epigenetics and the DNA methylation status, as well as the development of metabolic disorders [7,8]. Thus, DNA methylation and other epigenetic mechanisms could be a regulatory landscape that could explain (at least in part) the etiology of MetS. In this regard, some studies have found an association between DNA methylation and MetS or metabolic variables related to MetS [3,9,10]. 

Adipose tissue (AT), traditionally considered a mere energy storage depot, has been recently proposed as a central player in metabolism and its function is usually dysregulated in metabolic disorders and cancer [11,12]. A lot of recently published literature shows that deregulation of genes important in metabolism and adipose tissue biology can affect adipose tissue function and in turn the whole metabolic state [13,14,15,16,17,18,19,20,21,22,23,24,25,26,27,28,29,30,31]. Moreover, epigenetic deregulation has also been related to adipose tissue dysfunction, which can lead to metabolic disorders. In fact, our group has previously demonstrated that methylation levels of the LPL promoter are higher in the AT of MetS subjects, and these methylation levels are related to triglyceride withdrawal [3]. In agreement with our findings, it has been shown that dietary fatty acids can alter DNA methylation in AT [32].

Nevertheless, little is known about the association between MetS, global DNA methylation and the methylation status of genes related to the development of MetS in AT. Thus, the aim of this study was to analyze the overall DNA methylation state of visceral adipose tissue (VAT), as well as the DNA methylation at the promoter regions of specific genes related to adipogenesis (PPARG, PPARA), lipid metabolism (RXRA, SREBF2, SREBF1, SCD, LPL, LXRb), and inflammation (LRP1 C3, LEP, and TNF) in subjects with and without MetS.

## 2. Material and Methods

### 2.1. Study Population 

**Figure 1:**
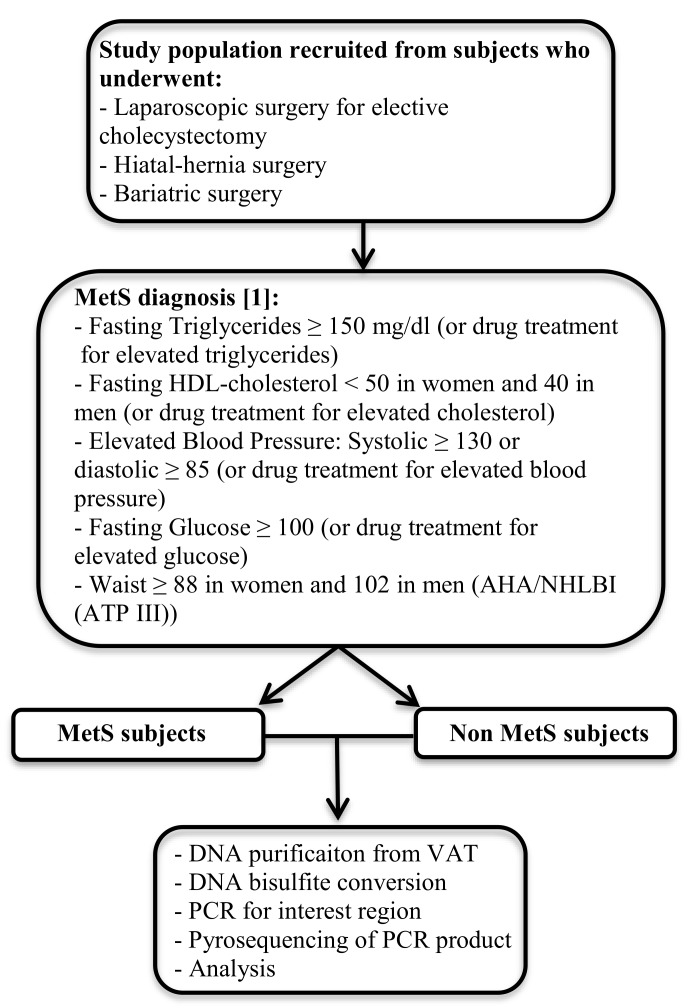
Diagram presenting the workflow of the study to measure global and specific DNA methylation levels.

This case-control study was performed in 55 patients without metabolic syndrome (Non MetS) and 53 patients with MetS recruited between January 2012 and December 2014 from the Endocrinology and Nutrition Department at Virgen de la Victoria Hospital (Malaga, Spain). MetS and Non MetS study subjects were recruited from patients that had undergone laparoscopic surgery for elective cholecystectomy, hiatal-hernia, or bariatric surgery. MetS patients were included if they met three or more of the updated parameters for the diagnosis of MetS according to Alberti’s Harmonization definition [1] and Non-MetS patients were selected if they had fewer than three MetS parameters (Figure 1). With respect to the drug treatment for blood pressure, lipids, and glucoses in MetS patients, 32% were treated with blood pressure lowering drugs, 11% with lipid lowering drugs and 3% with glucose lowering drugs, while in the case of the Non MetS population only 5% were treated with blood pressure lowering drugs.

Exclusion criteria in both study groups included the presence of cardiovascular disease, arthritis, acute inflammatory disease, infectious disease, or renal disease.

Study procedures included a comprehensive physical examination and blood analysis. Smoking habits and alcohol consumption were measured using a standardized questionnaire. 

The study was conducted in accordance with the guidelines laid down in the Declaration of Helsinki. All participants gave their written informed consent and the study was reviewed and approved by the Ethics and Research Committee of Virgen de la Victoria Hospital. 

### 2.2. Laboratory Measurements

Serum glucose, cholesterol, triglycerides (TG) and HDL cholesterol (HDL-c) were measured in a Dimension auto analyzer (Dade Behring Inc., Deerfield, IL, USA) by enzymatic methods (Randox Laboratories Ltd., Crumlin, UK). The LDL cholesterol (LDL-c) was calculated from the Friedewald equation. Insulin was quantified by radioimmunoassay (BioSource International, Camarillo, CA, USA). Serum leptin and adiponectin levels were analyzed by enzyme immunoassay (ELISA) kits (respectively: DSL, Webster, FL, USA; and DRG Diagnostics GmbH, Marburg, Germany). The homeostasis model assessment of insulin resistance (HOMA-IR) was calculated from fasting insulin and glucose with the following equation: HOMA-IR = fasting insulin (μIU/mL) × fasting glucose (mmol/L)/22.5 [33].

### 2.3. Visceral Adipose Tissue DNA Isolation and Pyrosequencing

VAT was obtained during laparoscopic surgery for elective cholecystectomy, hiatal-hernia or bariatric surgery. Biopsy samples were washed in physiological saline and were immediately frozen in liquid nitrogen. Biopsy samples were maintained at −80 °C until analysis. 

The PyroMark® Q96 ID Pyrosequencing System (Qiagen, Seoul, South Korea) was used to determine the methylation status of the study genes. The following pre-made assays (Qiagen, Valencia, CA, USA) were used: PPARA (PM00082635) (Figure S1), RXRA (PM00144431) (Figure S2), LXRb (PM00190260) (Figure S3), SREBF1 (PM00178087) (Figure S4), SREBF2 (PM00082516) (Figure S5), SCD (PM00042196) (Figure S6), LPL (PM00037401) (Figure S7), LRP1 (PM00051835) (Figure S8), C3 (PM00189399) (Figure S9), and LEP (PM00129724) (Figure S10). For the analysis of LINE-1 a tested assay which included 6 CpG sites was used [34]. We developed the assay for TNF and PPARG using the MethPrimer software [35]. 5 CpG sites were analyzed for TNF (Figure S11) while 5 CpG (Figure S12) sites were analyzed for PPARG as well using the following primer sets: TNF, Forward: GGAAAGGATATTATGAGTATTGAAAGTATG, Reverse: ACACTCACCTCTTCCCTCTAA, Sequencing primer: ATTATGAGTATTGAAAGTATGAT; PPARG: Forward: AAGAGGATTAGGTTTAGAATAGTATGT, Reverse: AATAAACAATAACCTTTTCTTTTCCTAC, Sequencing primer: AAGAGGGGTTTTAAGT. DNA methylation analyses were performed using bisulfite-treated DNA followed by a highly quantitative analysis based on PCR-pyrosequencing. The bisulfite conversion was done with 2 µg genomic DNA isolated from VAT using Qiazol (Qiagen, Valencia, CA, USA) and 0.1 µM citrate ethanol solutions. Then, the PCR was performed in 25 µL total volume, with a final primer concentration of 0.2 M. One of the primers was biotinylated in order to purify the final PCR product using sepharose beads. The biotinylated PCR amplifications were purified using the pyrosequencing Vacuum Prep-Tool (Qiagen, Valencia, CA, USA). Finally, 15 µL of the PCR products were pyrosequenced using the PyroMark Q96 ID Pyrosequencing System (Qiagen, Seoul, South Korea), and 0.4 µM sequencing primer.

The methylation level was expressed as the percentage methylated cytosine over the sum of methylated and unmethylated cytosines. Non-CpG cytosine residues were used as built-in controls to verify bisulfite conversion. The values were expressed as the mean for all the sites. We also included unmethylated and methylated DNA as controls in each run (New England Biolabs, Ipswich, MA, USA). Inter-assay precision (%CV) was <2.5%, intra-assay (%CV) was <1.0%.

### 2.4. Statistical Analysis

Comparisons of the anthropometric and biochemical characteristics as well as the DNA methylation levels between the study groups were made with a non-parametric test (Mann–Whitney U test). A variable named “MetS index” formed by the number of MetS components present in each subject, which ranged from 0 to 5, was defined. The Pearson’s correlation coefficients were calculated to evaluate the association between LINE-1 methylation and the anthropometric and biochemical variables. The Spearman’s correlation coefficients were calculated to evaluate the association between the specific-site methylation levels and the anthropometric and biochemical variables. Multiple linear regression analyses were performed to evaluate the contribution of diverse CpG DNA methylation variables to the changes in the levels of triglycerides (TG). We performed a linear regression analysis with TG as the dependent variable and with the CpG sites which were significant in the correlation study as independent variables, controlling for age and gender. Moreover, we tested through logistic regression what CpG dinucleotide could be a risk factor for MetS. This model was corrected for age and gender. Values were considered to be statistically significant when *p* < 0.05. The correlation and mean differences between groups analyzed were performed with SPSS (Version 15.0 for Windows; SPSS Iberica, Madrid, Spain). 

## 3. Results

### 3.1. Patient Characterization and Global Methylation

MetS patients showed a clear metabolic deterioration, with higher body mass index (BMI), waist circumference, glucose, insulin, HOMA-IR, TG, total cholesterol, LDL-c, apolipoprotein B (ApoB), systolic blood pressure (SBP), diastolic blood pressure (DBP) and leptin; and lower levels of HDL-c and adiponectin compared to the Non-MetS group (Table 1). 

In order to assess the global DNA methylation profile, DNA methylation at LINE-1 sequence was studied. Specifically, 6 CpG sites were included and pyrosequenced. The results showed no differences of global DNA methylation at any of the CpGs included between the study groups (Table 2).

We also performed a correlation analysis to determine the possible relationship between the global DNA methylation and the variables related to MetS (Table 3). We found a negative correlation between LINE-1 P2 and the MetS index. Furthermore, there were negative correlations between glucose levels and LINE-1 P1, P2 and P5.

### 3.2. Gene Specific DNA Methylation in MetS versus Non MetS

#### 3.2.1. Adipogenic and Lipid Metabolism Factors

**Figure 2:**
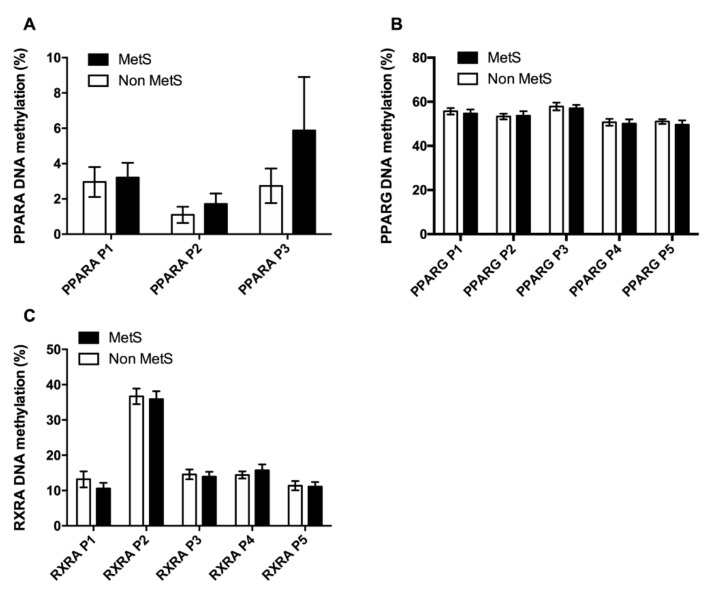
Adipogenic factors DNA methylation. DNA methylation profile across the CpG analyzed at the promoters of the adipogenic factors PPARA (**A**), PPARG (**B**), and the PPARs partner RXRA (**C**) in both, Non MetS and MetS groups. Values are given as the mean ± SE. Peroxisome proliferator-activated receptor alpha (PPARA); Peroxisome proliferator-activated receptor gamma (PPARG); Retinoid X receptor alpha (RXRA).

We studied genes related to adipose tissue development, such as PPARA, PPARG, and their heterodimer partner RXRA. As depicted in Figure 2, there were no differences in the methylation levels at any of the CpG sites analyzed for PPARA, PPARG or RXRA. Nevertheless, a tendency was observed for higher levels of DNA methylation in PPARA for MetS subjects than in Non MetS. 

In addition, we found a positive correlation between PPARA P2 with the MetS index, TG levels and HOMA-IR. PPARG P1 correlated positively with BMI, while PPARG P1 and P3 were negatively associated to DBP. In the case of the PPAR’s partner RXRA, we found a negative correlation between RXRA P1 and BMI and waist circumference.

**Figure 3:**
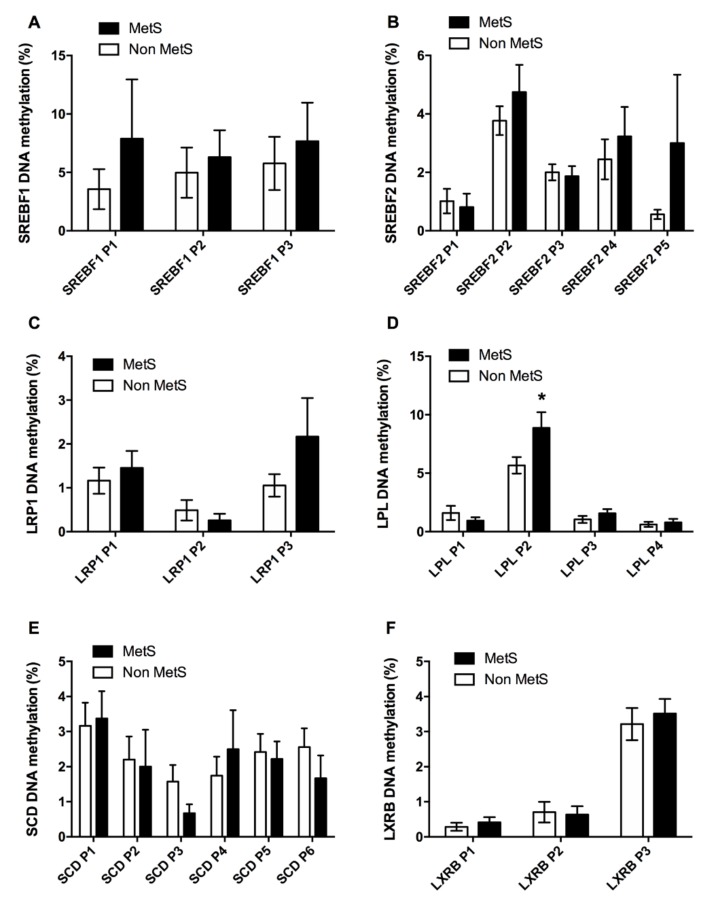
Lipid metabolism DNA methylation. The figure shows the DNA methylation in Non MetS and MetS groups at each CpG for several factors related to lipid metabolism as SREBF1 (**A**), SREBF2 (**B**), LRP1 (**C**), LPL (**D**), SCD (**E**), and LXRB (**F**). Values are given as the mean ± SE. Sterol regulatory element binding transcription factor 1 (SREBF1); Sterol regulatory element binding transcription factor 2 (SREBF2); low density lipoprotein receptor-related protein 1 (LRP1); lipoprotein lipase (LPL); Stearoyl-CoA desaturase (SCD); liver X receptor beta (LXRB). * means *p* < 0.05 according to a Student’s *T*-test.

A set of CpG sites inside gene promoters related only to lipid metabolism was also pyrosequenced. No differences were found at any of the CpGs analyzed for SREBF1, SREBF2 or LRP1 (Figure 3A–C). In the case of LPL, we found an increase in the DNA methylation levels for the CpG sites located at position 2 (LPL P2) (Figure 3D). We did not find different levels of DNA methylation at any of the CpG studied for the SCD or LXRB genes (Figure 3E,F). 

For these genes, we observed that the MetS index correlated negatively with SCD P6, while SCD P3 was negatively associated to BMI. Positive associations existed between TG levels and LPL P3, and between HDL-c and LRP1 P2. Furthermore, there was a negative association between the cholesterol regulator SREBF2 and DBP, specifically with SREBF2 P2. 

#### 3.2.2. Inflammation Factors

**Figure 4:**
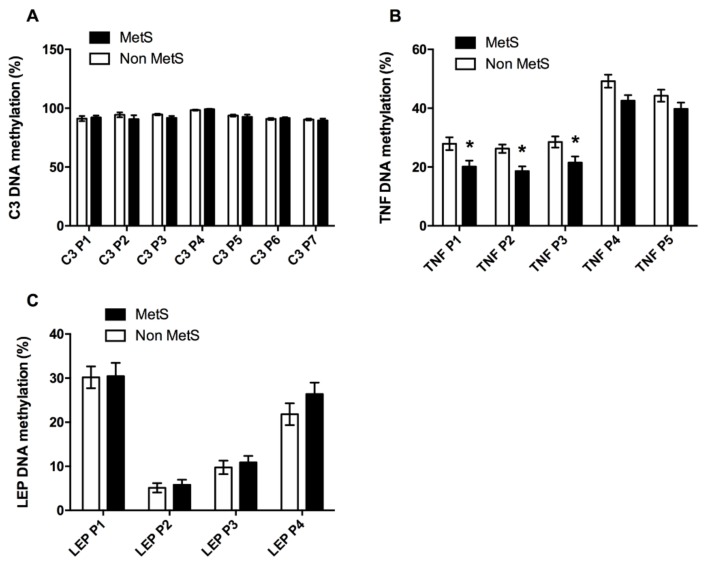
Inflammatory promoters DNA methylation. Comparisons between the Non MetS and the MetS group for DNA methylation at different CpG from genes implied in inflammatory processes as C3 (**A**), TNF (**B**) and LEP (**C**). Values are given as the mean ± SE. Complement factor 3 (C3); tumor necrosis factor (TNF); leptin (LEP). * means *p* < 0.05 according to a Student’s *T*-test.

Given the relationship between adipose tissue and inflammation in MetS, we analyzed some factors involved in this process. We analyzed 7 CpG sites inside the C3 gene promoter, but we did not find different DNA methylation levels between the Non MetS and the MetS subjects (Figure 4A). We also studied 5 CpG sites for the tumor necrosis factor (TNF). In this case, MetS subjects presented lower DNA methylation levels at 3 out of the 5 CpG sites that were analyzed, specifically at positions 1, 2 and 3 (TNF P1–P3) (Figure 4B). The third factor we studied was leptin (LEP), for which we analyzed 4 CpG sites at the leptin sequence, which did not present significant differences between Non MetS and MetS subjects (Figure 4C).

On the other hand, a negative relationship was found between the MetS index and the DNA methylation levels of TNF P2, TNF P3, TNF P4, and TNF P5 (Table 4). Glucose correlated negatively with the DNA methylation of TNF P4 (Table 4). For triglyceride levels, there were negative correlations found with TNF P2 and P5. Inversely to TG, HDL-c correlated positively with TNF P1, P2, P5 (Table 4). 

There was also a negative correlation between TNF P4 and LDL-c and DBP. Furthermore, there were positive and significant correlations between LEP P1 and LDL-c, SBP and DBP (Table 4). 

#### 3.2.3. Regression Analyses

To study the strength of the association observed in the correlation analyses we performed lineal regression analyses. We observed that the DNA methylation levels of PPARA P2 and LPL P3 could explain TG levels (Table 5), a regression that was corrected for age, gender, and BMI.

Furthermore, we performed a logistic regression analysis (harmonized by step method) to determine what factors could predict the risk of having MetS. We observed that TNF P2 remained as a protective variable, with a reduction of 23% in the probability of having MetS per unit of DNA methylation increase (Table 6).

## 4. Discussion

Epigenetic marks can be changed under developmental processes, nutritional conditions, exercise or metabolic status [36,37,38], highlighting the importance of epigenetics to fully understand the etiology of metabolic diseases. Altogether, our results show a general stability in the DNA methylation of the adipose tissue of our patients for the studied marks. The global methylation (LINE 1) remained stable between the study groups. However, some differences at specific genes were observed between groups. LPL and TNF genes were found to be especially affected, in line with our previous work where a difference in DNA methylation at the LPL promoter was described between subjects with and without MetS [3]. Even though the groups did not show great differences, the whole population showed interesting relationships with MetS components, something that may be indicative of the importance of the DNA methylation in metabolic disturbances. 

Epigenetic marks, and concretely DNA methylation, are receiving increased attention to explain the etiology of metabolic diseases. LINE-1 DNA methylation levels have been proposed as a measurement of the global DNA methylation of an individual [39]. We observed a negative relationship between the LINE-1 DNA methylation levels and the MetS index and serum glucose levels. This supports previous data where a negative association was found between LINE-1 methylation and the worsening of MetS, and especially the glucose levels in obese patients [9]. Furthermore, it has been shown that lower LINE-1 methylation in blood cells is related to an improvement in impaired glucose metabolism after a physical activity intervention in subjects with glucose metabolism disorders [40]. Thus, our study expands this inverse relationship between LINE-1 methylation and the worsening of MetS and glucose metabolism to a population with a different range of BMI and metabolic disorders.

Examining the master genes of metabolism and adipogenesis, PPARA is a factor that stimulates β-oxidation and that is the pharmacological target of a group of TG-lowering drugs called fibrates [41,42]. DNA methylation changes in PPARA have been related with metabolic worsening in rats [41]. We found a positive correlation between the PPARA DNA methylation level and the MetS index, TG levels and HOMA-IR, in line with previous results [41,42]. PPARG is the other nuclear hormone receptor superfamily member we studied. PPARG DNA methylation levels showed a negative association with DBP and, more interestingly, a positive correlation with BMI. It is known that PPARG mRNA levels are negatively associated with BMI, and that obese people have lower expression levels of this gene, which has been associated with adipose tissue dysfunction [43]. PPARG down-regulation leads to impaired capacity of adipose tissue to accumulate lipids, which in turn provokes ectopic lipid accumulation, insulin resistance and other obesity-associated comorbidities [44]. Thus, this association observed between PPARG methylation and BMI is in accordance with previous studies [43,44]. Interestingly, a previous study has shown an increase in PPARG methylation and a decrease of PPARG mRNA associated with this DNA methylation increase in db/db and diet-induced obesity mice [45], which is in line with our results. Moreover, PPARG methylation deregulation has been described in the context of colorectal cancer, hepatitis B and liver inflammation and fibrosis associated with hepatitis B [46,47,48].

Both, PPARA and PPARG receptors carry out their actions by a heterodimer formed with their partner RXRA [49]. We observed a negative correlation between adipose tissue RXRA methylation and the BMI and waist circumference. Contrary to this result, but in a non-metabolic tissue, RXRA hypermethylation in the umbilical cord has been associated with higher adiposity later in childhood, a methylation status dependent on maternal nutrition [50]. 

Dysfunctional lipid metabolism is characteristic of metabolic diseases. Obesity has been traditionally correlated to low levels of HDL-c [51]. LRP1 is a factor involved in lipid homeostasis and it has been shown to be overexpressed in adipose tissue in obesity and to control intracellular cholesterol storage and fatty acid synthesis [52,53]. We found a positive association between LRP1 DNA methylation and HDL-c. Moreover, MetS patients exhibited higher levels of LPL methylation. LPL methylation levels together with PPARA methylation levels were capable of explaining fasting TG levels. This was in line with a previous study carried out by our group, where this LPL methylation not only explained fasting TG levels but also postprandial TG [3]. In addition, we found a negative relationship between SCD DNA methylation and BMI and the MetS index. SCD is a key enzyme in the conversion of polyunsaturated fatty acids (PUFAS) to monounsaturated fatty acids (MUFAS), and it has been shown to be overexpressed in adipose tissue in obesity and metabolic disorders. The absence of SCD has been related with an improvement of metabolic syndrome features in mice [54]. Furthermore, a decrease in SCD DNA methylation has been associated with weight loss in both a dietary intervention study and after bariatric surgery [55,56].

Adipose tissue inflammation, which can be the response of adipose tissue to overnutrition, is well documented in obesity and metabolic disorders. This inflammation can trigger a deterioration of the adipose tissue and in turn the metabolic state, which as a consequence reinforces the inflammatory process [57]. In this sense, we noted lower levels of TNF DNA methylation in MetS subjects compared to Non MetS subjects. We also found negative correlations between TNF methylation levels and the MetS index, BMI, TG, glucose, LDL-c and DBP, and a positive association with HDL-c. Thus, TNF methylation seems to be an epigenetic mark highly affected by MetS parameters, and is a risk factor for MetS according to our data. In line with our results, lower levels of adipose tissue TNF methylation have been described in diabetic subjects [58]. Besides, TNF methylation together with LEP DNA methylation in adipose tissue is capable of predicting responsiveness to a low-calorie diet [59]. Adipose tissue TNF is almost entirely produced by the macrophage fraction [60], and it is a gene highly expressed in M1 phenotype macrophages [61]. Moreover, in obesity and adipose tissue dysfunction there is a higher macrophage infiltration, and a polarization from the M2 phenotype to the pro-inflammatory M1 phenotype [60,62], a process that is epigenetically regulated [61,63,64,65]. Therefore, the lower levels of TNF methylation in MetS could be a sign of the macrophage cellularity in the tissue. 

Lastly, LEP, a hormone secreted by adipose tissue known for exerting satiety, has been associated with pro-inflammatory processes as well [66]. LEP has been observed to stimulate a blood pressure rise in a process involving the sympathetic nervous system and kidney Na+ reabsorption. Leptin-resistance associated with obesity might, though, be prompting the related blood pressure increase [67,68]. We observed a positive relationship between LEP methylation and blood pressure, which at first would not agree with previous results unless due to a compensatory mechanism trying to re-establish lower leptin levels. Moreover, it is known that leptin can regulate cholesterol ester (CE) metabolism by activating the Hormone-sensitive Lipase (HSL) in macrophages, an enzyme that carries out the breakdown of CE, therefore protecting against atherosclerosis [69]. A positive association has also been shown between LEP methylation and LDL-c, both in blood cells and subcutaneous adipose tissue, in morbid obese subjects, whereas no association was found in visceral adipose tissue [70]. Our study showed a positive correlation between LEP DNA methylation in visceral adipose tissue and LDL-c in the whole study population. 

This study has as limitations the number of factors studied and its cross-sectional nature that does not allow us to infer causality, and that the duration of MetS was not measured. Nevertheless, as strengths it provides an exploratory insight for the epigenetic state for some important factors involved in human adipose tissue function and that we found to be related to MetS and metabolic worsening. 

## 5. Conclusions

In conclusion, we provide data supporting the idea that global DNA methylation and methylation at specific genes related to adipogenesis, lipid metabolism and inflammation in VAT are related to the etiology of MetS. We show that LINE-1 was positively associated with glucose. LPL and PPARA DNA methylation were strongly associated with TG levels, while TNF DNA methylation was associated with TG, glucose, HDL-c and blood pressure and SCD was negatively associated with MetS worsening. Finally, TNF DNA methylation could be an important factor in preventing MetS occurrence according to logistic regression analysis. Our study shows the importance of better understanding the epigenetic regulation of adipose tissue in order to widen the possible role of this tissue in the etiology of the MetS, which could lead to new therapeutic epigenetic strategies.

## Figures and Tables

**Table 1:** Biochemical and anthropometric parameters in non-metabolic syndrome subjects (Non MetS) and metabolic syndrome subjects (MetS).

	Non MetS (*n* = 55)	MetS (*n* = 53)
Age (years)	48.4 ± 13.9	52.7 ± 14.6
Male/female (%)	52/48	44/56
BMI (Kg/m^2^) **	29.8 ± 7.9	36.4 ± 10.9
WC (cm) **	97.6 ± 14.8	112.6 ± 22.4
Glucose (mg/dL) **	94.3 ± 11.6	118.4 ± 29.5
Insulin (pmol/L) **	9.8 ± 7.4	16.2 ± 11.4
HOMA-IR **	2.3 ± 1.9	4.7 ± 3.4
TG (mg/dL) **	101.6 ± 38.1	164.2 ± 65.1
Cholesterol (mg/dL) **	194.0 ± 32.5	214.5 ± 41.3
HDL-c (mg/dL) **	55.0 ± 11.0	48.5 ± 14.2
LDL-c (Friedwald) *	119.0 ± 31.8	135.1 ± 30.2
ApoA1 (mg/dL)	171.6 ± 21.8	160.5 ± 29.5
ApoB (mg/dL) **	91.9 ± 22.3	108.7 ± 22.3
SBP (mm Hg) **	123.5 ± 17.8	139.8 ± 19.5
DBP (mm Hg) **	76.1 ± 11.2	82.7 ± 10.3
GOT (mg/dL)	20.0 ± 13.1	19.3 ± 8.7
GPT (mg/dL)	40.3 ± 23.9	44.6 ± 21.5
GGT (mg/dL)	57.4 ± 203.4	42.1 ± 27.9
Uric acid (mg/dL) **	4.6 ± 1.2	5.6 ± 1.2
Leptin (ng/mL) **	18.9 ± 23.8	38.1 ± 30.5
Adiponectin (μg/mL) *	11.2 ± 5.3	8.2 ± 4.1

Body mass index (BMI), waist circumference (WC), homeostatic model assessment of insulin resistance (HOMA-IR), baseline triglycerides (TG), high-density lipoprotein cholesterol (HDL-c), low density lipoprotein cholesterol (LDL-c), apolipoprotein A1 (ApoA1), Apolipoprotein B (ApoB), systolic blood pressure (SBP), diastolic blood pressure (DBP), glutamate-oxaloacetate transaminase (GOT), glutamate-pyruvate transaminase (GPT), gamma glutamyl transpeptidase (GGT). * *p* < 0.05 and ** *p* < 0.01 considered statistically significant according to a Student’s *T*-test and chi-squared test for gender.

**Table 2:** DNA methylation level for each CpG position at LINE-1 pyrosequenced in both Non MetS and MetS groups. Values are given as the mean ± SE.

	Non MetS	MetS
LINE-1 P1 (%)	74.15 ± 0.39	74.37 ± 0.34
LINE-1 P2 (%)	65.84 ± 0.20	65.75 ± 0.33
LINE-1 P3 (%)	55.21 ± 0.29	55.29 ± 0.29
LINE-1 P4 (%)	61.37 ± 0.33	61.48 ± 0.24
LINE-1 P5 (%)	65.02 ± 0.17	65.24 ± 0.21
LINE-1 P6 (%)	65.05 ± 0.48	64.71 ± 0.23

Non metabolic syndrome group (Non MetS); metabolic syndrome group (MetS); long interspersed element 1 (LINE-1).

**Table 3:** Pearson’s correlation between LINE-1 CpG positions (P1, P2, P3, P4, P5, P6) and the anthropometric and biochemical variables related to MetS. * *p* < 0.05 and ** *p* < 0.01 were considered statistically significant.

	MetS Index	BMI	Waist	Glucose	Tg	HDL-c	LDL-c	SBP	DBP	HOMA-IR
LINE-1 P1	−0.167	0.057	−0.031	−0.246 *	−0.088	0.113	0.082	0.162	0.02	−0.114
LINE-1 P2	−0.233 *	0.025	−0.068	−0.334 **	−0.208	0.074	0.028	0.171	0.010	−0.199
LINE-1 P3	−0.136	0.018	−0.011	−0.168	−0.072	−0.115	0.093	0.220	0.155	−0.101
LINE-1 P4	−0.068	0.042	0.012	−0.158	0.039	−0.112	0.077	0.168	0.010	−0.041
LINE-1 P5	−0.137	0.093	−0.037	−0.238 *	0.016	−0.139	0.05	0.136	0.100	−0.088
LINE-1 P6	−0.19	−0.055	−0.05	−0.137	−0.166	0.028	0.052	0.066	0.016	−0.126

Number of metabolic syndrome variables present in the subject of study (MetS index); body mass index (BMI); triglycerides (TG); high-density lipoprotein cholesterol (HDL-c); low-density lipoprotein cholesterol (LDL-c); systolic blood pressure (SBP); diastolic blood pressure (DBP); homeostatic model assessment of insulin resistance (HOMA-IR); long interspersed element 1 DNA methylation at positions 1 to 6 (LINE-1 P1–P6). * and ** mean *p* < 0.05 and *p* < 0.01 respectively according to Pearson’s correlation.

**Table 4:** Correlation analyses between anthropometric and biochemical variables associated to MetS with some of the DNA methylation at the CpG islands (CpGi) analyzed. Only CpG that presented any significant association are represented.

	MetS Index	BMI	Waist	Glucose	Tg	HDL-c	LDL-c	SBP	DBP	HOMA-IR
PPARA P2	0.276 *	0.076	0.165	0.166	0.392 **	0.061	0.08	0.066	−0.025	0.229 *
PPARG P1	−0.072	0.306 *	0.169	−0.224	−0.194	0.015	−0.197	−0.2	−0.293 *	−0.058
PPARG P3	−0.078	0.138	0.174	0.03	−0.139	0.021	−0.218	0.037	−0.283 *	0.112
RXRA P1	−0.102	−0.298 **	−0.229 *	−0.052	0.025	−0.095	0.127	−0.066	−0.225	−0.032
SREBF2 P2	0.056	0.006	0.144	0.112	0.136	−0.032	0.189	−0.224	−0.262 *	0.121
LRP1 P2	0.09	−0.065	−0.048	0.114	−0.215	0.373 *	−0.055	0.192	0.180	0.251
LPL P3	0.135	0.029	0.089	0.128	0.245 *	−0.102	0.085	0.126	−0.111	0.149
SCD P3	−0.056	−0.340 *	−0.283	−0.096	−0.018	0.108	0.22	0.087	−0.117	−0.03
SCD P6	−0.325 *	−0.116	−0.17	−0.141	−0.134	0.121	0.102	−0.275	−0.232	−0.172
TNF P1	−0.212	0.132	0.046	−0.034	−0.188	0.283 *	−0.02	−0.010	−0.115	0.029
TNF P2	−0.420 **	0.054	−0.061	−0.192	−0.273 *	0.304 *	−0.195	−0.188	−0.217	−0.196
TNF P3	−0.320 *	0.151	−0.021	−0.094	−0.155	0.222	−0.109	−0.237	−0.242	−0.03
TNF P4	−0.330 *	−0.006	−0.096	−0.278 *	−0.203	0.098	−0.295 *	−0.245	−0.305 *	−0.133
TNF P5	−0.281 *	0.132	−0.100	−0.153	−0.281 *	0.380 **	−0.132	−0.097	−0.008	−0.074
LEP P1	0.088	0.081	−0.159	0.061	−0.071	0.015	0.229 *	0.264 *	0.230 *	0.028

Number of metabolic syndrome variables present in the subject of study (MetS index); body mass index (BMI); Triglycerides (TG); high-density lipoprotein cholesterol (HDL-c); low-density lipoprotein cholesterol (LDL-c); systolic blood pressure (SBP); diastolic blood pressure (DBP); homeostatic model assessment of insulin resistance (HOMA-IR); peroxisome proliferator-activated receptor alpha DNA methylation at position 2 (PPARA P2); retinoid X receptor alpha methylation at position 1 (RXRA P1); leptin DNA methylation at position 1 (LEP P1); sterol regulatory element binding transcription factor DNA methylation at position 2 (SREBF2 P2); Stearoyl-CoA desaturase DNA methylation at positions 3 and 6 (SCD P3 and P6); Tumor necrosis factor DNA methylation at positions P1 to P5 (TNF P1–P5); peroxisome proliferator-activated receptor gamma DNA methylation at positions 1and 2 (PPARG P1 and P2); Lipoprotein lipase DNA methylation at position 3 (LPL P3); Low-density lipoprotein receptor-related protein 1 DNA methylation at position 2 (LRP1 P2). * and ** means *p* < 0.05 and *p* < 0.01 respectively.

**Table 5:** Lineal regression analysis with fasting triglycerides as dependent variable and PPARA P2, LPL P3, and TNF P2 as independent variables and corrected for age, gender and BMI.

	Fasting triglycerides (*R* = 0.566; *R*^2^ = 0.320)		
	β	P	CI 95%
Age	0.111	0.425	−0.582–1.358
Gender	−0.268	0.047	−48.377–(−0.312)
BMI	−0.101	0.446	−1.825–0.816
PPARA P2	0.332	0.012	1.32–10.012
LPL P3	0.264	0.046	0.099–10.72
TNF P2	−0.117	0.347	−1.867–0.669

Body mass index (BMI); peroxisome proliferator-activated receptor alpha DNA methylation at position 2 (PPARA P2); lipoprotein lipase DNA methylation at position 3 (LPL P3); tumor necrosis factor DNA methylation at position 2 (TNF P2).

**Table 6:** Logistic regression analysis: risk of MetS. The regression was conducted using a step method to look for the most harmonized model. Variables that showed a significant association with the MetS index at the correlation analyses, such as Age, gender, PPARA P2, SCD P6, and TNF P2 to P5, were introduced as independent variables and a harmonized model in which gender, PPARA P2, and TNF P2 were maintained was generated.

	Non Mets/MetS (*R*^2^ = 0.506–0.686)		
	OR	*p*	CI 95%
Gender	5.813	0.094	0.739–45.699
PPARA P2	1.630	0.246	0.714–3.719
TNF P2	0.791	0.008	0.664–0.942

Non metabolic syndrome group (Non MetS); Metabolic syndrome group (MetS); Peroxisome proliferator-activated receptor alpha DNA methylation at position 2 (PPARA P2); Tumor necrosis factor DNA methylation at position 2 (TNF P2).

## Supplementary Materials

The following are available online at http://www.mdpi.com/2077-0383/8/1/87/s1, Figure S1: Genomic region overview of the promoter of PPARA gene; Figure S2: Genomic region overview of the promoter of RXRA gene; Figure S3: Genomic region overview of the promoter of LXRB gene; Figure S4: Genomic region overview of the promoter of SREBF1 gene; Figure S5: Genomic region overview of the promoter of SREBF2 gene; Figure S6: Genomic region overview of the promoter of SCD gene; Figure S7: Genomic region overview of the promoter of LPL gene; Figure S8: Genomic region overview of the promoter of LRP1 gene; Figure S9: Genomic region overview of the promoter of C3 gene; Figure S10: Genomic region overview of the promoter of LEP gene; Figure S11: Genomic region overview of the promoter of TNF gene; Figure S12: Genomic region overview of the promoter of PPARG gene.

## Abbreviations

MetS	Metabolic syndrome
Non MetS	Non metabolic syndrome
CpGi	CpG island
VAT	Visceral adipose tissue
TG	Triglycerides
HOMA-IR	homeostasis model assessment of insulin resistance
BMI	Body mass index
LDL-c	LDL cholesterol
HDL-c	HDL cholesterol
SBP	Systolic blood pressure
DBP	Diastolic blood pressure
PUFAS	Monounsaturated fatty acids
PPARG	Peroxisome Proliferator Activated Receptor Gamma
PPARA	Peroxisome Proliferator Activated Receptor Alpha
RXRA	Retinoid X receptor alpha
SREBF1	Sterol regulatory element-binding transcription factor 1
SREBF2	Sterol regulatory element-binding transcription factor 2
SCD	Stearoyl-CoA desaturase
LXRB	Liver X receptor beta
LRP1	Low density lipoprotein receptor-related protein 1
C3	Complement component 3
TNF	Tumoral necrosis factor
LEP	Leptin
