# Supplementary material for: Altered Adipose Tissue DNA Methylation Status in Metabolic Syndrome: Relationships Between Global DNA Methylation and Specific Methylation at Adipogenic, Lipid Metabolism and Inflammatory Candidate Genes and Metabolic Variables
Source: J Clin Med. 2019 Jan 13;8(1):87. doi: 10.3390/jcm8010087 (PMC6352101; doi:10.3390/jcm8010087)
Supplement: Supplementary file 1 [file jcm-08-00087-s001.pdf]

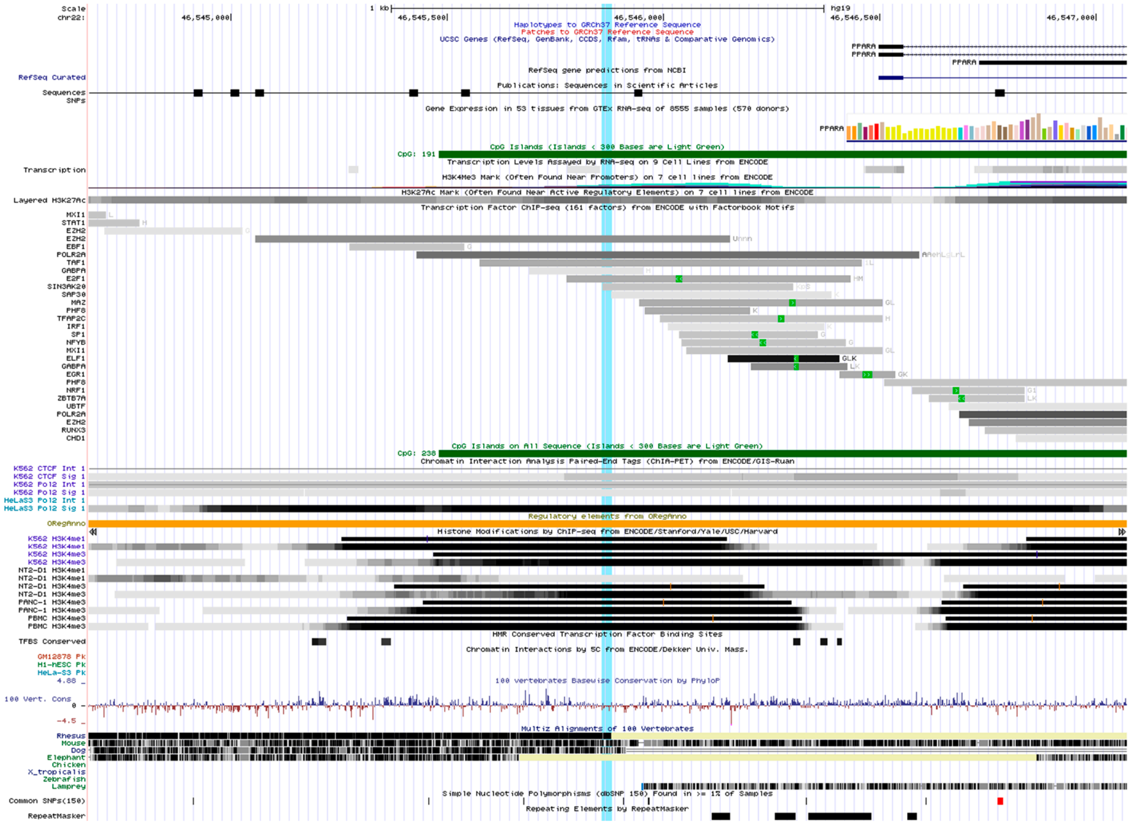

Figure S1. Genomic region overview of the promoter of PPARA gene where the assay was performed (highlighted in light blue). The overview shows the transcriptions factor which has been demonstrated to be bound to the region by in vivo experiment (ENCODE project). The image was extracted from GRCh37/hg39 assembly of UCSC Genome Browser.

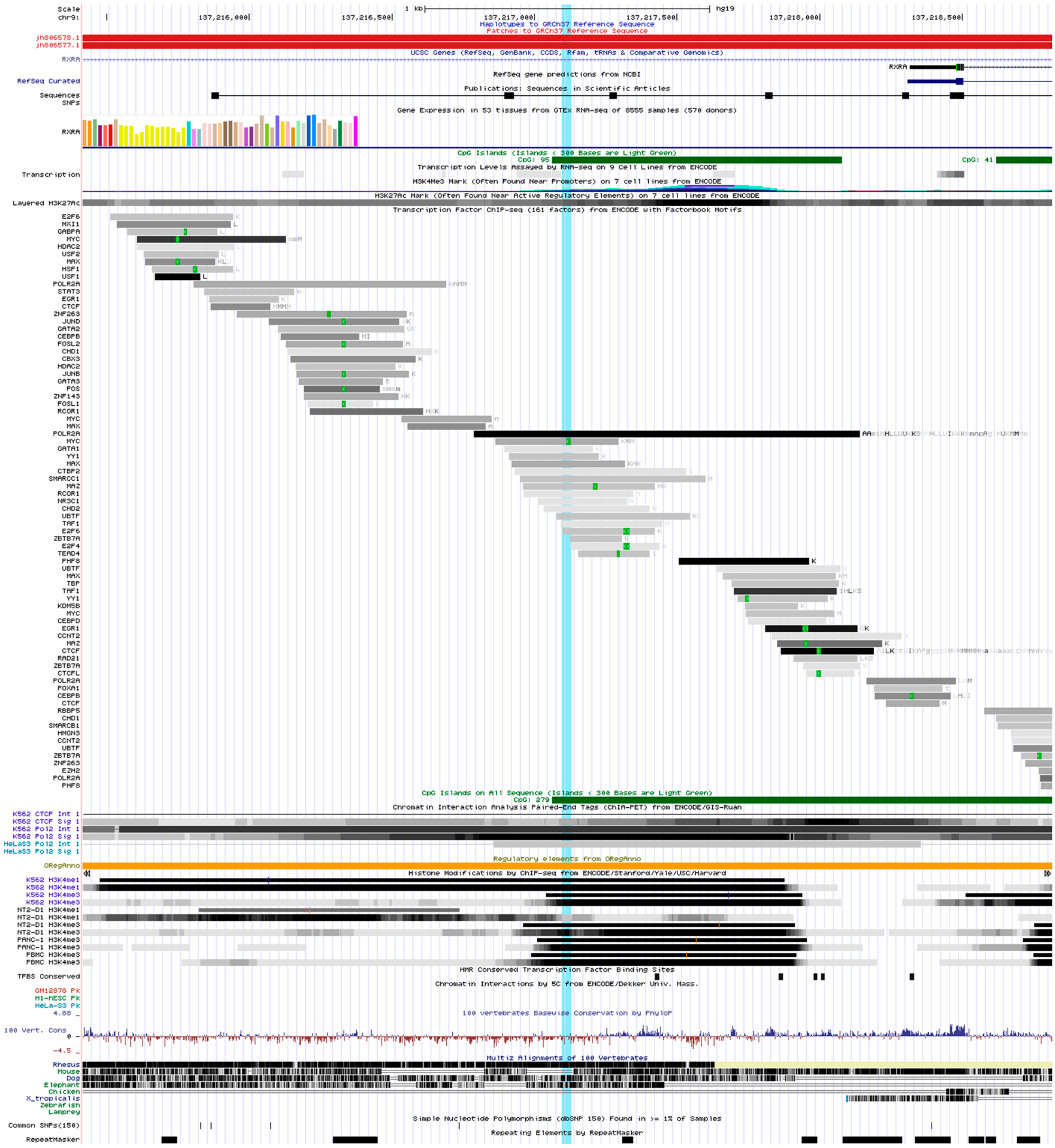

Figure S2. Genomic region overview of the promoter of RXRA gene where the assay was performed (highlighted in light blue). The overview shows the transcriptions factor which has been demonstrated to be bound to the region by in vivo experiment (ENCODE project). The image was extracted from GRCh37/hg39 assembly of UCSC Genome Browser.

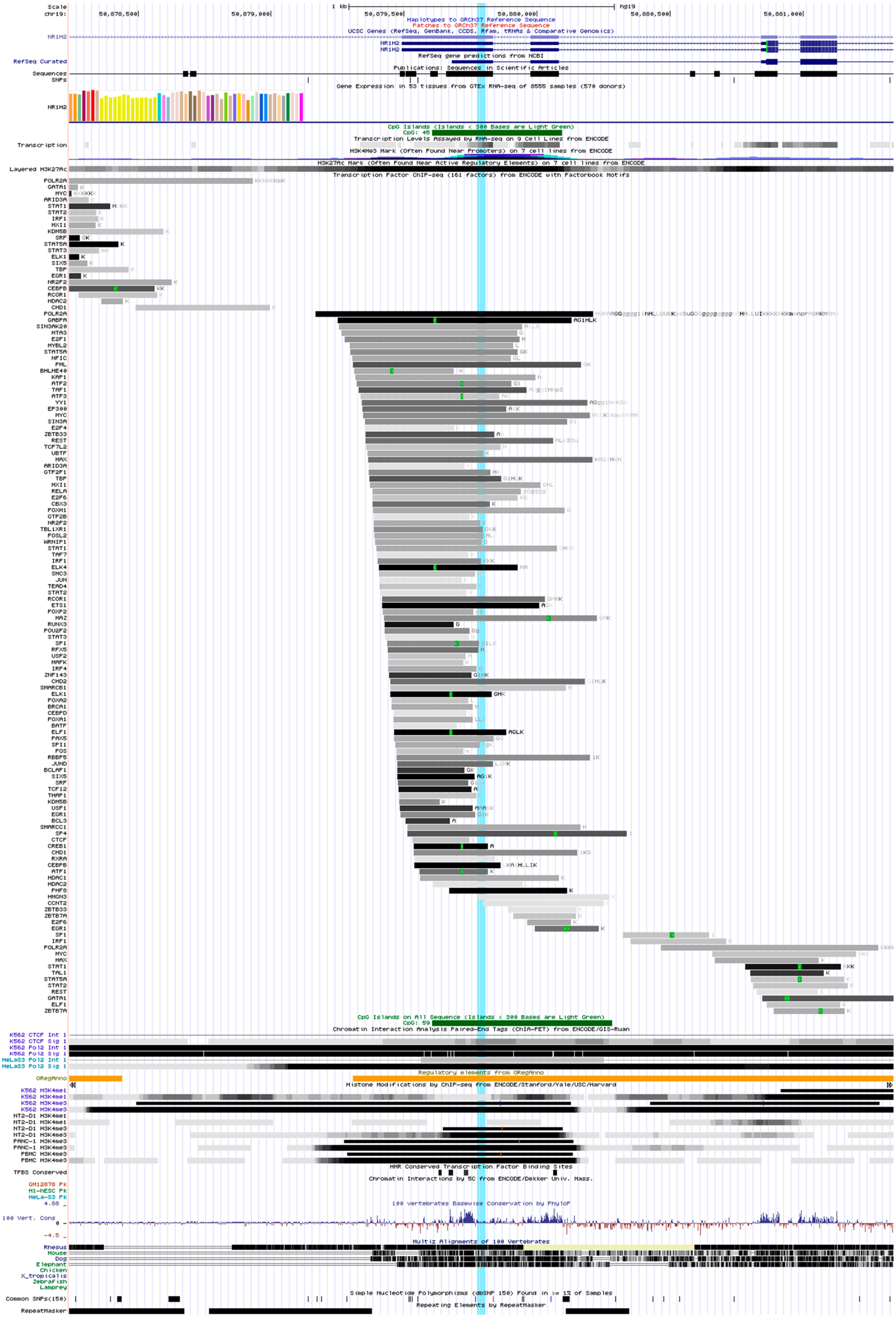

Figure S3. Genomic region overview of the promoter of LXRB gene where the assay was performed (highlighted in light blue). The overview shows the transcriptions factor which has been demonstrated to be bound to the region by in vivo experiment (ENCODE project). The image was extracted from GRCh37/hg39 assembly of UCSC Genome Browser.

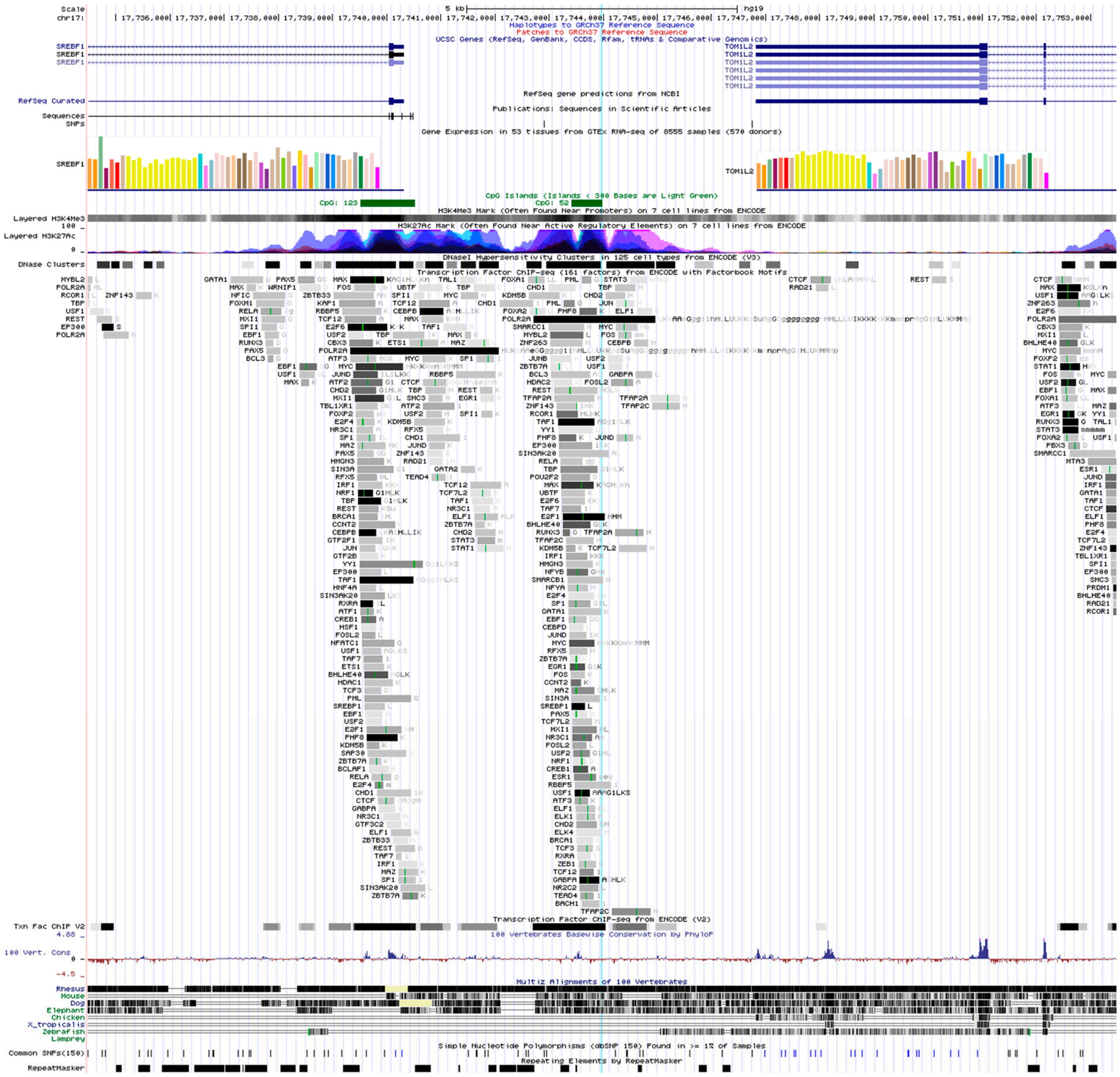

Figure S4. Genomic region overview of the promoter of SREBF1 gene where the assay was performed (highlighted in light blue). The overview shows the transcriptions factor which has been demonstrated to be bound to the region by in vivo experiment (ENCODE project). The image was extracted from GRCh37/hg39 assembly of UCSC Genome Browser.

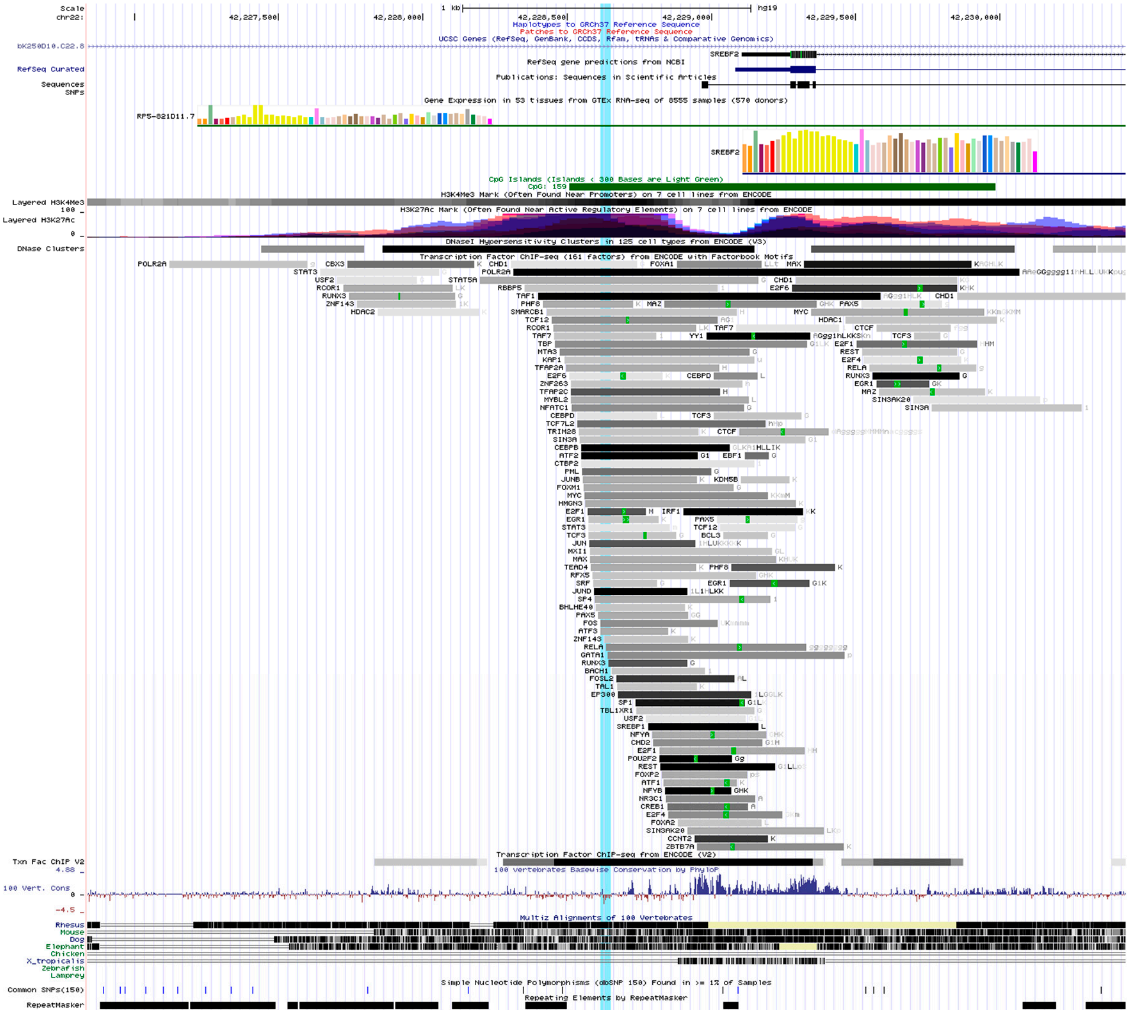

Figure S5. Genomic region overview of the promoter of SREBF2 gene where the assay was performed (highlighted in light blue). The overview shows the transcriptions factor which has been demonstrated to be bound to the region by in vivo experiment (ENCODE project). The image was extracted from GRCh37/hg39 assembly of UCSC Genome Browser.

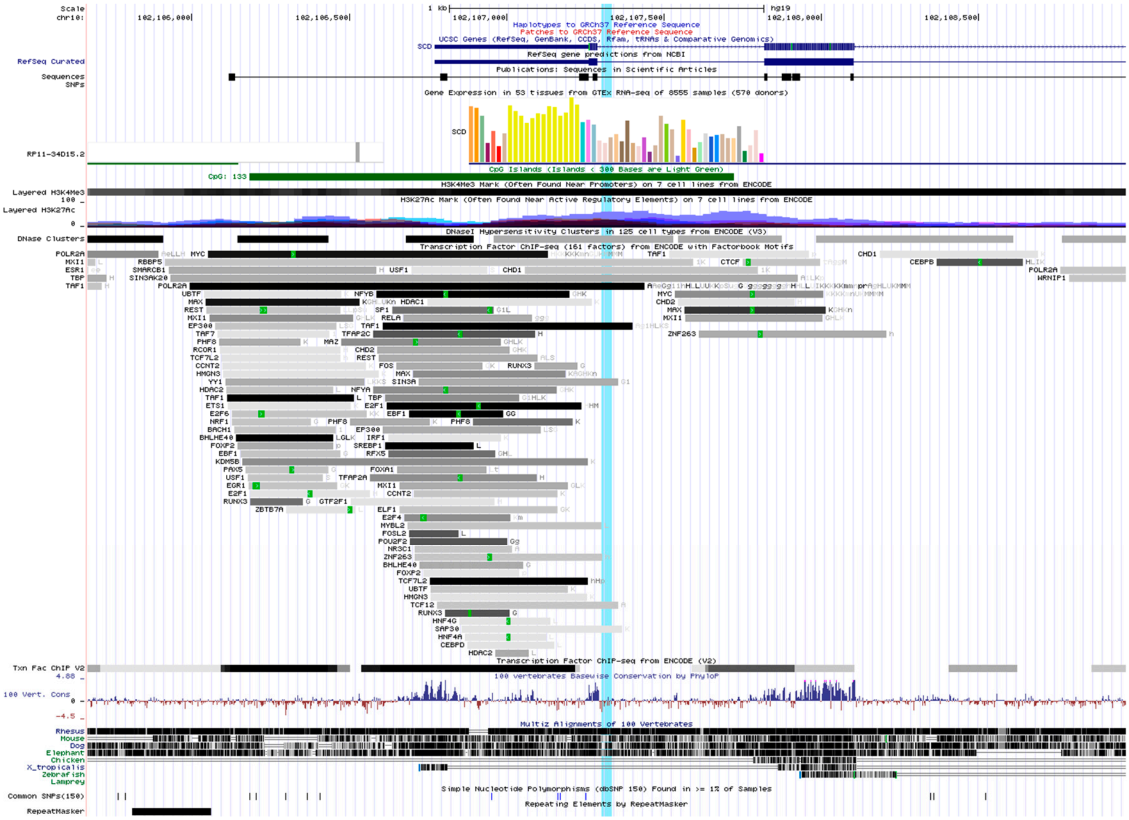

Figure S6. Genomic region overview of the promoter of SCD gene where the assay was performed (highlighted in light blue). The overview shows the transcriptions factor which has been demonstrated to be bound to the region by in vivo experiment (ENCODE project). The image was extracted from GRCh37/hg39 assembly of UCSC Genome Browser.

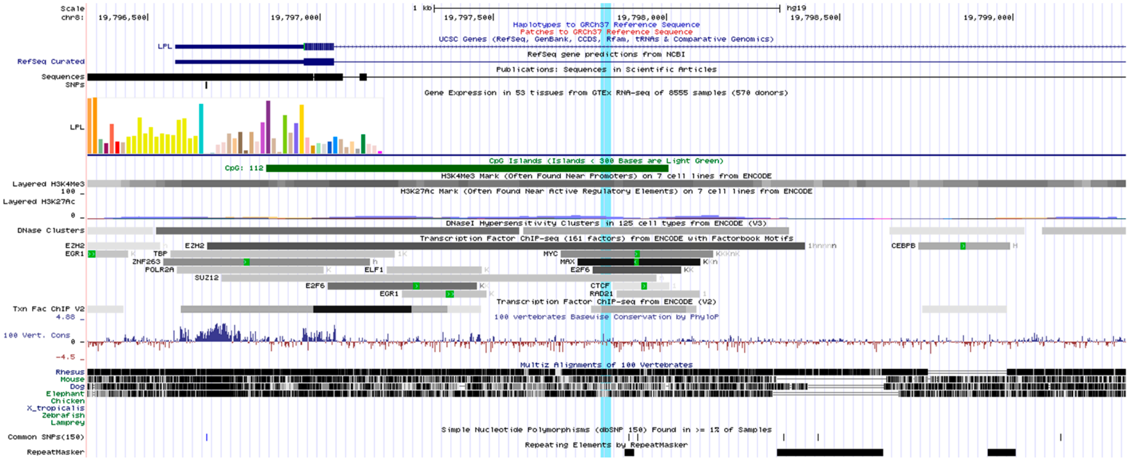

Figure S7. Genomic region overview of the promoter of LPL gene where the assay was performed (highlighted in light blue). The overview shows the transcriptions factor which has been demonstrated to be bound to the region by in vivo experiment (ENCODE project). The image was extracted from GRCh37/hg39 assembly of UCSC Genome Browser.

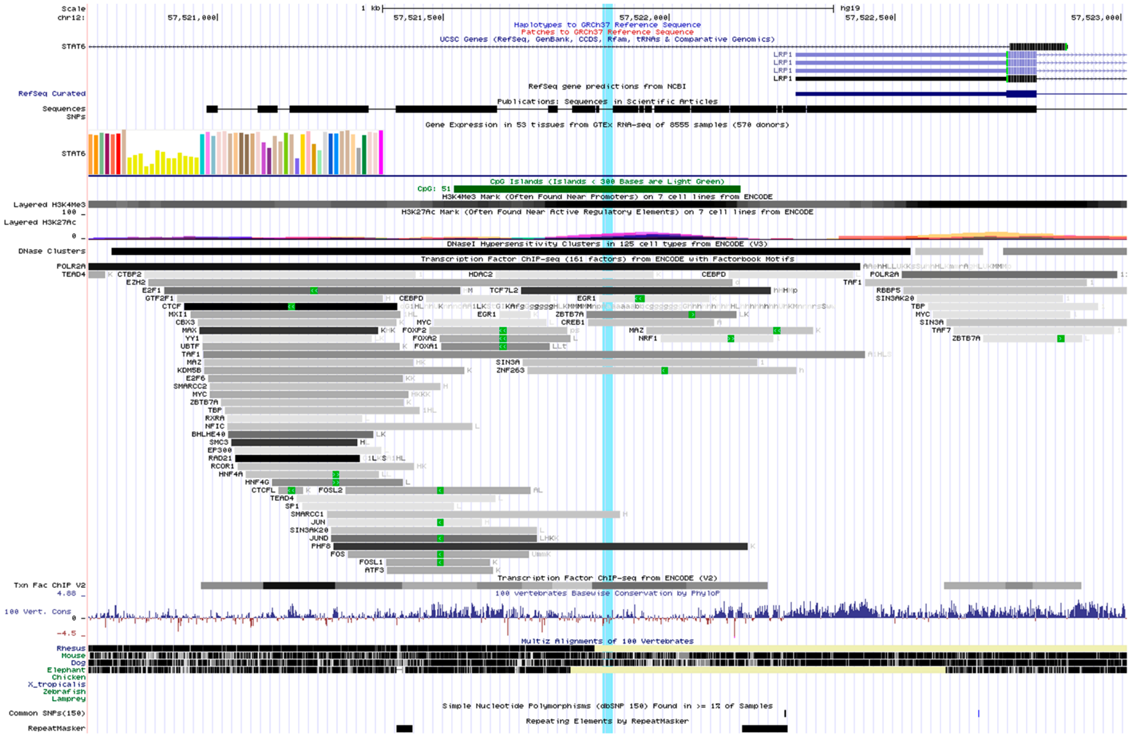

Figure S8. Genomic region overview of the promoter of LRP1 gene where the assay was performed (highlighted in light blue). The overview shows the transcriptions factor which has been demonstrated to be bound to the region by in vivo experiment (ENCODE project). The image was extracted from GRCh37/hg39 assembly of UCSC Genome Browser.

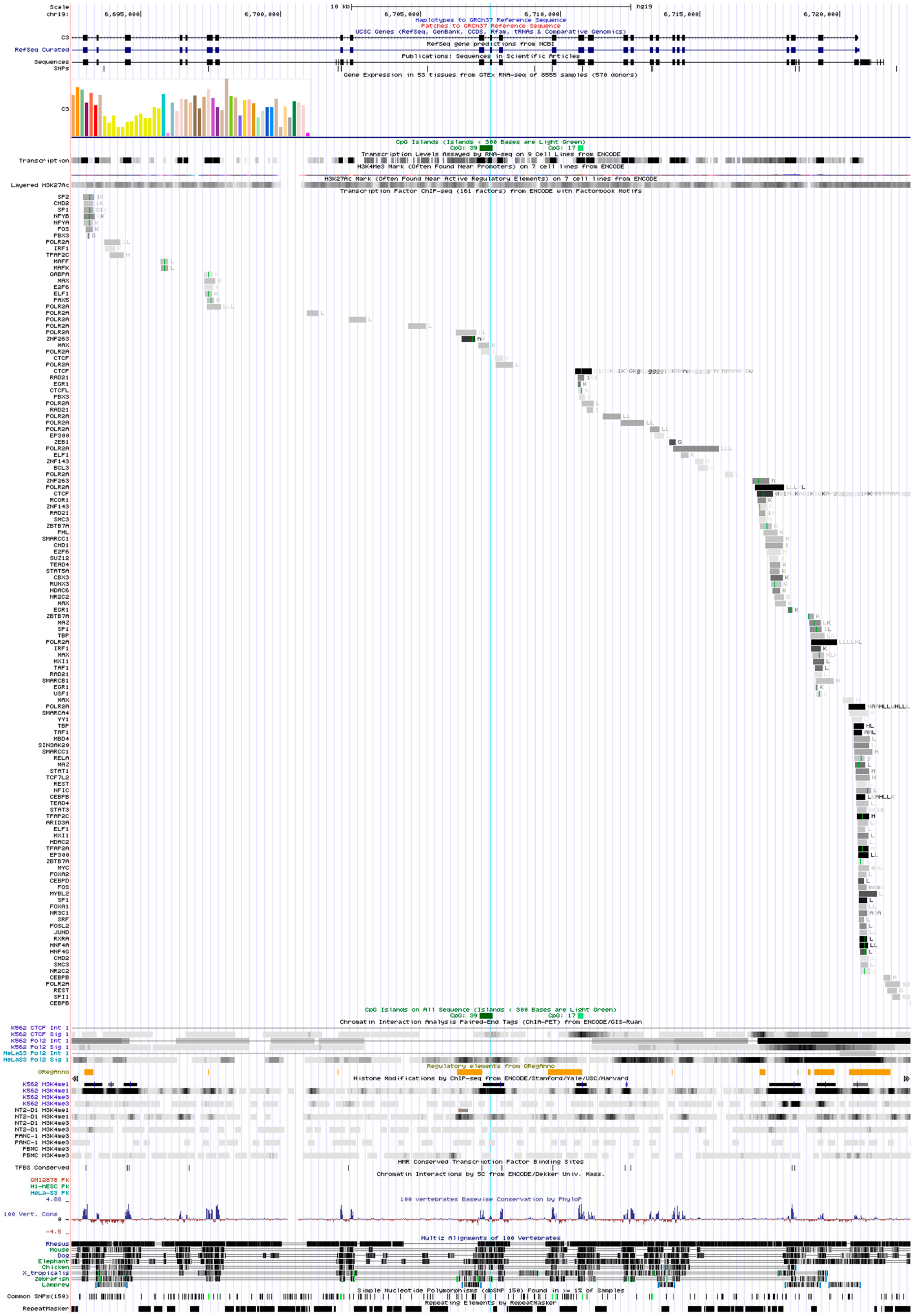

Figure S9. Genomic region overview of the promoter of C3 gene where the assay was performed (highlighted in light blue). The overview shows the transcriptions factor which has been demonstrated to be bound to the region by in vivo experiment (ENCODE project). The image was extracted from GRCh37/hg39 assembly of UCSC Genome Browser.

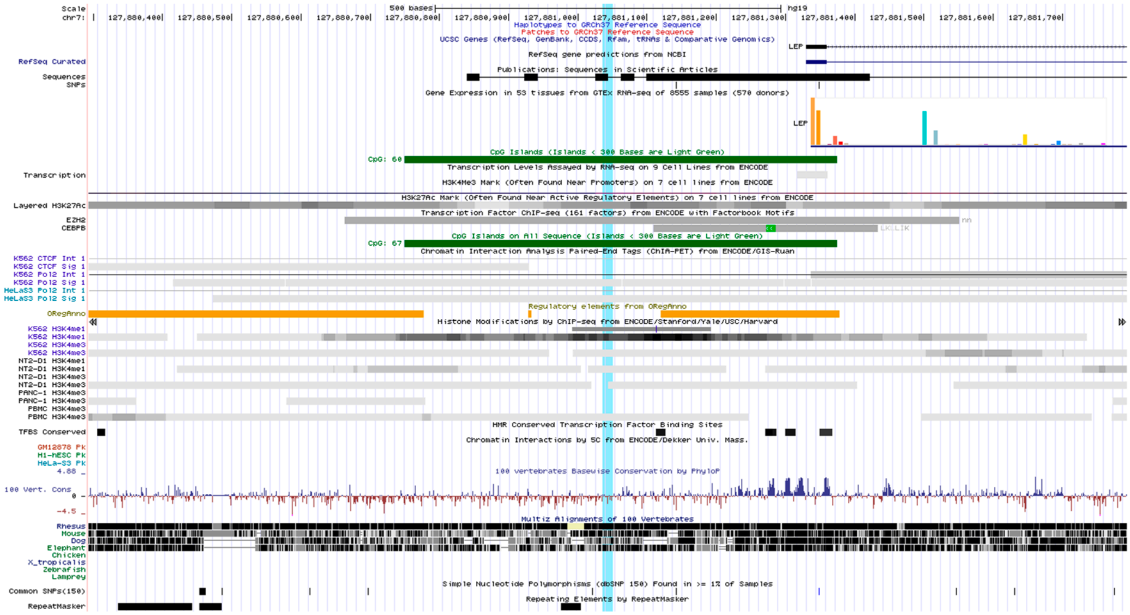

Figure S10. Genomic region overview of the promoter of LEP gene where the assay was performed (highlighted in light blue). The overview shows the transcriptions factor which has been demonstrated to be bound to the region by in vivo experiment (ENCODE project). The image was extracted from GRCh37/hg39 assembly of UCSC Genome Browser.

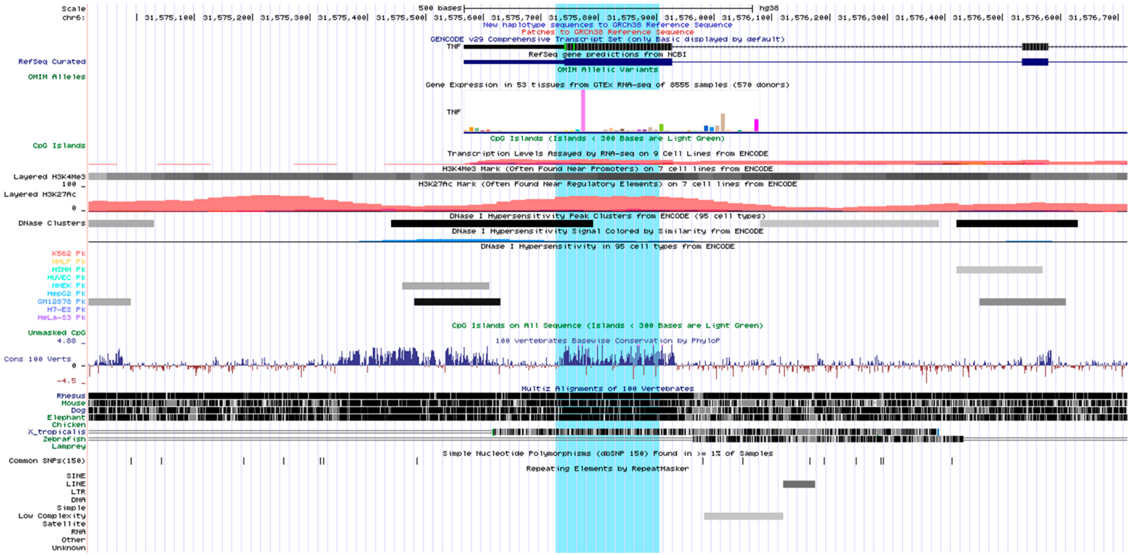

Figure S11. Genomic region overview of the promoter of TNF gene where the assay was performed (highlighted in light blue). The overview shows the transcriptions factor which has been demonstrated to be bound to the region by in vivo experiment (ENCODE project). The image was extracted from GRCh37/hg39 assembly of UCSC Genome Browser.

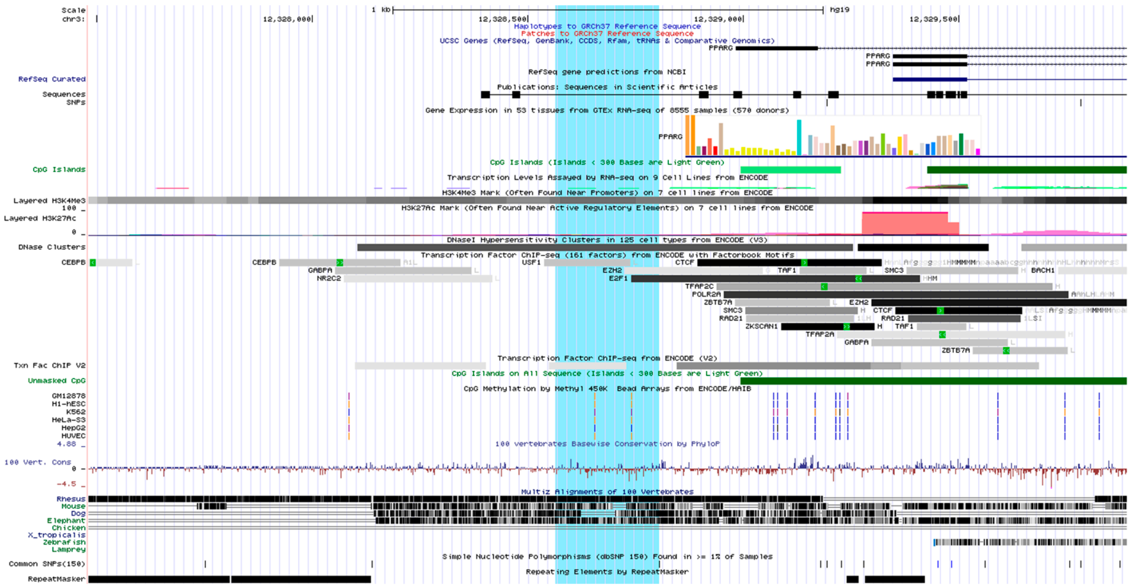

Figure S12. Genomic region overview of the promoter of PPARG gene where the assay was performed (highlighted in light blue). The overview shows the transcriptions factor which has been demonstrated to be bound to the region by in vivo experiment (ENCODE project). The image was extracted from GRCh37/hg39 assembly of UCSC Genome Browser.
